# Interventions to prevent the onset of frailty in adults aged 60 and older (PRAE-Frail): a systematic review and network meta-analysis

**DOI:** 10.1007/s41999-024-01013-x

**Published:** 2024-07-26

**Authors:** Annette Eidam, Jane Durga, Jürgen M. Bauer, Samuel Zimmermann, Johannes A. Vey, Kilian Rapp, Michael Schwenk, Matteo Cesari, Petra Benzinger

**Affiliations:** 1 Center for Geriatric Medicine, Heidelberg University Hospital, Agaplesion Bethanien Hospital Heidelberg, Rohrbacher Straße 149, 69126 Heidelberg, Germany; 2 Network Aging Research (NAR), Heidelberg University, Heidelberg, Germany; 3 Institute of Medical Biometry, Heidelberg University, Heidelberg, Germany; 4 Department of Clinical Gerontology, Robert-Bosch-Hospital, Stuttgart, Germany; 5 Department of Sport Science, Human Performance Research Centre, University of Konstanz, Konstanz, Germany; 6 Department of Clinical Sciences and Community Health, University of Milan, Milan, Italy; 7 Faculty of Social and Health Studies, Institute of Health and Generations, University of Applied Sciences Kempten, Kempten, Germany

**Keywords:** Frailty prevention, Pre-frailty, Older adults, Geriatrics

## Abstract

**Purpose:**

Frailty in older adults is associated with multiple adverse health outcomes, while evidence on its successful prevention has been scarce. Therefore, we analyzed the effectiveness of different interventions for the prevention of frailty onset.

**Methods:**

In this systematic review, eight databases were searched for randomized controlled trials of interventions in non-frail (i.e., robust or pre-frail) adults aged ≥ 60 years that assessed frailty incidence at follow-up. Additive component network meta-analysis (CNMA) was conducted to isolate the effect of different intervention types on the main outcome of frailty incidence, reporting relative risk (RR) with 95% confidence intervals (CI). The effect on gait speed was analyzed as an additional outcome using a classic network meta-analysis and the standardized mean difference (SMD) with 95% CI.

**Results:**

We screened 24,263 records and identified 11 eligible trials. Nine trials (842 participants, all categorized according to the physical phenotype) in pre-frail (seven RCTs) and robust/pre-frail (two RCTs) older adults were included in the CNMA. Physical exercise significantly reduced frailty incidence at follow-up (RR 0.26, 95% CI 0.08; 0.83), while this was not found for nutritional interventions (RR 1.16, 95% CI 0.33; 4.10). Interventions based on physical exercise also improved gait speed (SMD 1.55, 95% CI 1.16; 1.95). In addition, 22 eligible trial protocols without published results were identified.

**Conclusion:**

Interventions based on physical exercise appear to be effective in preventing the onset of frailty in older adults. Although the available data are still limited, results from ongoing trials may add to the body of evidence in the foreseeable future.

**Supplementary Information:**

The online version contains supplementary material available at 10.1007/s41999-024-01013-x.

## Key summary points

**Aim:**

To analyze the effectiveness of different interventions in preventing frailty onset in older adults.

**Findings:**

In this network meta-analysis, interventions that were based on physical exercise significantly reduced frailty incidence.

**Message:**

Physical exercise appears to be effective in the prevention of frailty onset in older adults.

**Supplementary Information:**

The online version contains supplementary material available at 10.1007/s41999-024-01013-x.

## Introduction

Frailty is associated with adverse outcomes such as falls, hospitalization, disability, and mortality [1] and it affects older adults’ autonomy and quality of life [2, 3]. Approximately 10% of community-dwelling adults aged ≥ 65 years are frail, and frailty prevalence in the community setting increases to 15.7% in adults aged 80–84 years and to 26.1% in adults aged ≥ 85 years [4]. Due to the high burden of frailty in an aging society, policymakers are showing an increasing interest in this geriatric syndrome to improve the health care of the aging population [5].

Most experts agree that a dynamic continuum can be described from robustness to frailty with a transitional, intermediate state termed “pre-frailty” [6]. Transitions between frailty states are common [7, 8]. In an observational study of community-dwelling adults aged ≥ 70 years, more than 50 percent experienced a worsening or an improvement of their frailty status over a period of 4.5 years [7]. Systematic reviews provide evidence that a targeted treatment of frailty is possible, as demonstrated by a reduction in frailty status and a delay in frailty progression [9–11].

Considering the importance of frailty on both an individual and societal level, it is indicated to not only address the therapy of frailty but also its prevention. However, evidence-based recommendations for older adults, health care practitioners, and policymakers for the prevention of frailty have yet to be developed. Just recently, a systematic review of clinical practice guidelines for frailty prevention and management reported a substantial lack of evidence-based guidance in this regard [12]. Two comprehensive network meta-analyses (NMA) have evaluated the effectiveness of different intervention types in the management of frailty [10, 13]. Yet, a comparable NMA that exclusively targets the context of frailty prevention in robust and pre-frail older adults is still missing.

To fill this gap, we performed a systematic review and network meta-analysis (NMA) to synthesize the evidence from randomized controlled trials (RCTs) and thereby assess the effect of different interventions in preventing the onset of frailty in robust or pre-frail older adults. Our aim is to present the currently available evidence with the intention to support the development of future recommendations for the prevention of this detrimental condition.

## Methods

This systematic review is reported according to the Preferred Reporting Items for Systematic Reviews and Meta-Analyses (PRISMA) extension statement for reporting of systematic reviews incorporating network meta-analyses of health care interventions (see online supplementary Table 1) [14]. The protocol for this research was registered on PROSPERO (Registration number CRD42020208067).

### Eligibility criteria

#### Types of studies

We included RCTs—either individual or cluster-randomized—that were reported in a peer-reviewed full-text article.

#### Types of participants and frailty assessments

This review included trials in adults aged ≥ 60 years. In case age-related eligibility was not reported, studies with a mean or median age of ≥ 65 years (in all study arms) were also considered for inclusion. Participants had to be non-frail, i.e., robust or pre-frail, at trial baseline according to the results of a valid assessment instrument. The list of 25 frailty instruments considered in this systematic review (see online supplementary Table 2) was derived from a recent review and a practice guideline [15, 16]. Cut-points for frailty were identified from the literature. The different frailty instruments were ranked, and their hierarchy was based on the number of frailty domains (e.g., physical, psychosocial, or pharmacological) covered by each frailty instrument. In case multiple eligible frailty instruments were used in a given study, the participants’ frailty status was determined using the highest-ranked frailty instrument that was reported. In case no ranges for the respective frailty instruments were available, study populations with a mean ± two standard deviations or a 95% confidence interval (CI) or an interquartile range below the frailty cut-point at baseline were considered as non-frail. Trials with mixed non-frail and frail populations were only included if separate results for the non-frail group were reported. Trials conducted primarily in the inpatient hospital setting were excluded, while all other settings were eligible. Trials only considering participants with specific diseases were excluded, except for certain conditions highly prevalent in older adults (sarcopenia, mild cognitive impairment, malnutrition, obesity, and osteopenia).

#### Types of interventions

Any type of intervention as well as any combination of interventions to prevent the onset of frailty was considered for inclusion. However, we excluded trials with pharmacological interventions such as drug trials or trials that focused primarily on medication management. Trials with medication management as part of a multi-domain intervention were eligible for inclusion. Safety considerations were used to distinguish between pharmacological and nutritional interventions. Nutrients (e.g., vitamins) given at doses above the daily Tolerable Upper Intake Level set by the European Food Safety Authority (EFSA) were defined as pharmacological interventions. For interventions using vitamin D, we considered studies using doses ≤ 2000 IU per day as eligible. For other dietary components (e.g., caffeine), we referred to estimates of safe intake published by the EFSA to define eligibility. Interventions related to advanced care planning or palliative care were also excluded.

#### Types of comparators

We considered studies with control arms without any intervention/with usual care or control arms with minimally or likely ineffective interventions.

#### Types of outcome measures

Studies that reported frailty as an outcome measure using a valid frailty instrument (see online supplementary Table 2) were eligible for inclusion. Because we were interested in frailty incidence as the main outcome, frailty outcomes had to be reported in a binary mode using the respective cut-points (see online supplementary Table 2). For instance, studies using the physical phenotype of frailty were required to report how many participants scored above or below the frailty cut-point of 3 fulfilled criteria at follow-up, while studies using gait speed as their highest-ranked frailty outcome measure had to report how many participants had a gait speed of < 0.8 m/s (frail) or ≥ 0.8 m/s (non-frail) at follow-up. Additional outcomes included additional frailty outcome measures, measures of activities of daily living (ADLs), instrumental ADLs (IADLs), health-related quality of life, physical activity, adherence, and adverse events.

#### Eligibility of study protocols

To give a narrative synthesis of ongoing or recently completed trials that had not yet published results but might potentially fit the eligibility criteria for this systematic review on frailty prevention, we also included full-texts of trial protocols, i.e., published full-text articles that described the design of a trial without presenting comprehensive results. Protocols were eligible if they reported on RCTs, used a valid frailty instrument at trial baseline and follow-up, met the eligibility criteria for types of interventions and types of comparators, and did not obviously violate the age-related and/or frailty-status-related inclusion criteria. As soon as a full-text article reporting comprehensive results of the RCTs presented was identified, we removed the respective protocol from our list of eligible study protocols.

### Information sources

Eight databases were searched for published articles: Cochrane Library databases, PubMed, CINAHL, PEDro, Web of Science (included Science Citation Index-EXPANDED), PsycINFO, Embase, and BiblioMap. The search was conducted between November 11, 2021 (PubMed) and February 10, 2022 (Embase), and all databases were searched from their respective inception. No language restrictions were applied during the search. Authors of eligible study protocols that were published before 2021 were contacted in September 2022 about available publications of main results related to the respective RCTs. We did not contact the authors if the recruitment for the trial was reported to be ongoing at that time. In December 2023 we conducted an update search based on the full-text articles of eligible trial protocols that had been identified during the original screening and selection process. Using the trial registration numbers of the RCTs presented in these protocols, we searched for newly published full-text articles of main trial results on the PubMed database, within the respective trial registries, and on Google Scholar.

### Search strategy

In order to detect all valid frailty instruments, the search strategy contained “frail*” or the individual name of the frailty instrument. The search strategy for the PubMed search is shown in online supplementary Table 3.

### Selection of studies

After the removal of duplicates, titles and abstracts were screened independently by two reviewers (JD, PB) using the software Covidence (www.covidence.org). The selection of studies was restricted to languages of the European Union at this stage. Full texts were then assessed for eligibility independently by two reviewers (JD, AE, or PB). In the case of disagreement, conflicts were solved by discussion or by consulting with the third reviewer.

### Data extraction

A data extraction sheet was developed using Covidence. Data items from the relevant publications were extracted independently by two reviewers (JD, AE) for all eligible studies and by one author (JD or AE) for eligible study protocols. Reviewers resolved disagreements by discussion or by consulting with a third reviewer (PB). Information retrieved included, amongst others, characteristics of the trial (e.g., study design, country, registration number), characteristics of the interventions (type and duration of intervention in the different study arms, setting in which the intervention was delivered), characteristics of the participants (e.g., number of participants randomized to and analyzed in each treatment arm, baseline characteristics of participants per treatment arm including frailty status), and primary and secondary outcome measures. In individual cases, authors of the included studies were contacted to ask for clarifying information related to the primary frailty outcome measure.

### Risk of bias assessment

Risk of bias of individual trials for the frailty outcome was assessed independently by two reviewers (JD, AE) using the Cochrane risk-of-bias tool for randomized trials version 2 (RoB2) [17]. Conflicts were solved by discussion or by consulting with a third assessor (PB).

### Statistical analysis and data synthesis

For the main outcome of frailty onset (binary), treatment effects are expressed as risk ratios (RR) between an intervention and the corresponding control group and reported alongside a 95% CI. For the calculation of the RR, the continuity correction (adding 0.5) was applied for study arms with zero events. The additional outcome of gait speed (continuous) was analyzed for studies that reported it. The Standardized Mean Differences (SMD) alongside a 95% CI were used as summary measure to account for different scales used in the individual studies. For studies that reported mean and standard deviation of gait duration for a specified distance instead of gait speed, mean gait speed was calculated by dividing distance by mean gait duration. The corresponding standard deviations were calculated using the Taylor approximation. Further, two arms (“Exercise slow”/”Exercise fast”) in Coelho-Júnior [18] were summarized according to Cochrane guidelines to create a single “Exercise”-arm to be included in the analysis.

To analyze the research question presented, NMA was conducted to estimate treatment effects compared to the baseline effect of a combined control group (placebo, usual care, minimally or likely ineffective control intervention). Because some interventions present in the included studies are combinations of others, component network meta-analysis (CNMA) [19] was conducted in an attempt to isolate the specific treatment effects for every single type of intervention, which is called a component. Assuming additive effects for combinations of components implying that the treatment effect of a combination is the sum of the effects of its components, the additive CNMA model was applied with random effects since clinical heterogeneity between trials was expected. Comparisons belonging to multi-arm studies were accounted for by reweighting the respective multi-arm studies [20, 21]. Additionally, a conventional NMA was conducted where each existing single intervention or combination was considered as a separate node in the network. The frequentist method based on graph theory for data synthesis [20] was employed and the τ^2^ and I^2^ –statistics were used to assess between-trial variance. Heterogeneity and inconsistency were quantified by the *Q* statistic [22]. The additivity assumption was examined by the difference of the *Q* statistic between the additive CNMA and the standard NMA model. Network graphs for all conducted analyses are provided to visualize the connectedness of the related networks alongside tables containing the resulting estimated effects for each NMA. A subgroup analysis containing only studies with comparatively short treatment protocols (≤ 16 weeks) was conducted to assess the impact of intervention duration on the overall effect. This should also be interpreted as a supporting sensitivity analysis. Where applicable, forest plots are used to illustrate the estimated results of the two modelling approaches (conventional NMA versus additive CNMA) against one another to detect discrepancies. A comparison-adjusted funnel plot was created to assess publication bias by means of funnel plot asymmetry in an NMA [23].

All analyses were performed in R [24] (version 4.2.2) using the packages meta (version 6.1.0) [25] and netmeta (version 2.7.0) [26].

## Results

### Study selection

**Fig. 1:**
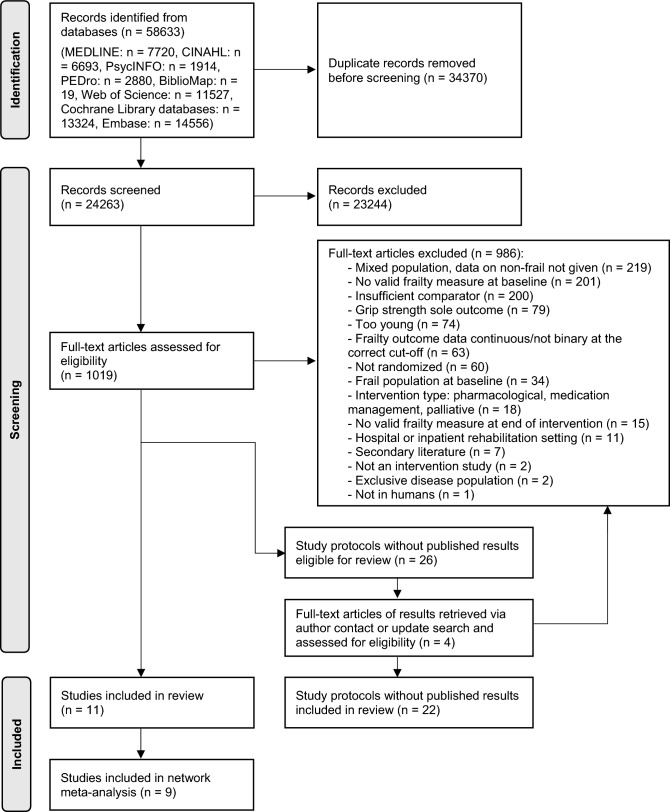
PRISMA flow diagram of the screening and selection process. Adapted from [27]

After the removal of duplicates, 24,263 titles and abstracts and then 1019 full-text articles were screened for eligibility. A total of 11 trials were retained for the present analyses: nine of these were included in the NMA and an additional two studies were included in a qualitative summary. In addition, 26 eligible study protocols were identified. One full-text article reporting trial results for one of these protocols was received through author contact and three full-text articles reporting results for three of the trials presented in the protocols were identified during the update search in December 2023. However, none of these full-text articles met the eligibility criteria. The PRISMA flow diagram of the screening and selection process is shown in Fig. 1.

### Characteristics of included studies

The characteristics of the eleven included trials are presented in Table 1. The studies were conducted in seven different countries, and all studies were individually randomized parallel RCTs. The number of participants analyzed for frailty ranged from 32 to 252 per trial. In nine trials, the physical phenotype of frailty proposed by Fried and colleagues was the main frailty-related outcome measure, while five of these nine trials reported using a modified version of this frailty instrument. One trial administered a Comprehensive Geriatric Assessment (CGA) and one trial the Groningen Frailty Indicator to assess frailty. Six of the eleven studies included only non-frail older adults, while five studies had a mixed population and reported separate results for their non-frail participants.

**Table 1:** Characteristics of included trials

Author, year, country, study design	Main frailty assessment	Population	Sample size	Mean age [years]	Male [%]	Type of intervention	Intervention protocol	Duration of intervention	Relevant timepoints of frailty assessment
Badrasawi et al. [28], 2016 Malaysia Parallel RCT	Physical phenotype	Pre-frail	N randomized (IG) 29 (CG) 29 Total: 58 N analyzed^a^ (IG) 26 (CG) 24 Total: 50	(IG) 68.2 (CG) 68.8	(IG) 53.8 (CG) 37.5	IG: nutrition	IG: l-carnitine 500 mg TID CG: placebo	10 weeks	Baseline 10 weeks
Barrachina-Igual et al. [29], 2021 Spain Parallel RCT	Physical phenotype	Pre-frail	N randomized (IG) 27 (CG) 23 Total: 50 N analyzed^a^ (IG) 23 (CG) 20 Total: 43	(IG) 74.8 (CG) 75.3	(IG) 30.4 (CG) 25.0	IG: exercise	IG: two ~ 65 min exercise sessions per week (10 min warm-up, progressive high-intensity resistance training, self-massage for myofascial release, cool-down) CG: routine daily activities	12 weeks	Baseline (one week before start of intervention) 14 weeks (one week after completion of intervention)
Biesek et al. [30], 2021 Brazil Parallel RCT	Physical phenotype	Pre-frail	N randomized^a^ (IG1) 18 (IG2) 18 (IG3) 18 (IG4) 18 (CG) 18 Total: 90 N analyzed (IG1) 15 (IG2) 18 (IG3) 16 (IG4) 15 (CG) 15 Total: 79	(IG1) 71.2 (IG2) 73.1 (IG3) 71.7 (IG4) 69.7 (CG) 70.4	(IG1) 0 (IG2) 0 (IG3) 0 (IG4) 0 (CG) 0	IG1: exercise IG2: nutrition IG3: exercise + nutrition IG4: exercise + placebo	IG1: two supervised exercise sessions per week (exergames with a balance platform and a weighted vest; ~ 10 min warm-up, ~ 10 min neuromotor exercises, ~ 20 min progressive resistance exercises, ~ 10 min cool-down) IG2: nutritional supplement (171 kcal, 21 g whey protein, 5 g lipids, 224 mg calcium, 3.3 µg vitamin D, 23 mg vitamin C, 2.3 g leucine, 12 g essential amino acids) taken 5 days per week IG3: exercise + nutritional supplement IG4: exercise + isoenergetic placebo CG: normal daily activities	12 weeks	Baseline 12 weeks
Chen et al. [31], 2020 China Parallel RCT	(Modified) Physical phenotype	Pre-frail	N randomized (IG) 35 (CG) 35 Total: 70 N analyzed^a^ (IG) 33 (CG) 33 Total: 66	(IG) 77.0 (CG) 75.3	(IG) 36.4 (CG) 33.3	IG: exercise	IG: three 45–60 min group exercise session per week (warm-up, elastic band exercises, cool-down) CG: normal daily activities	8 weeks	Baseline 8 weeks
Coelho-Júnior & Uchida [18], 2021 Brazil Parallel RCT	(Modified) Physical phenotype	Pre-frail (The study included also a frail group; both groups were randomized separately)	N randomized (IG1) 13 (IG2) 13 (CG) 13 Total: 39 N analyzed^a^ (IG1) 11 (IG2) 11 (CG) 10 Total: 32	(IG1) 65 (IG2) 65 (CG) 65	(IG1) 18.2 (IG2) 0 (CG) 0	IG1: exercise slow IG2: exercise fast	IG1 + IG2: group exercise sessions; four-week familiarization period with four lower limb resistance exercises, then 12-week main exercise period performing the same four exercises wearing weight vests and ankle weights during low-speed resistance training (IG1) or high-speed resistance training (IG2) CG: one 20 min flexibility session per week	16 weeks	Baseline 16 weeks
Gené Huguet et al. [32], 2018 Spain Parallel RCT	(Modified) Physical phenotype	Pre-frail	N randomized^a^ (IG) 100 (CG) 100 Total: 200 N analyzed (IG) 85 (CG) 88 Total: 173	(IG) 84.5 (CG) 84.5	(IG) 32.0 (CG) 39.0	IG: multi-domain (medication review, nutrition, exercise, social)	IG: (1) Medication review with STOPP/START criteria in participants with polypharmacy (≥ 5 drugs); recommendation of treatment changes to individual family physician. (2) Group session with expert on Mediterranean diet, who gave advice on individual nutritional changes. (3) Exercise: (i) aerobic exercise (walking 30–60 min a day ≥ 3 days per week) and (ii) exercises for strength, resistance, balance, and coordination (nine fortnightly sessions at primary healthcare center and 3–4 days per week at home). (4) Assessment by social worker (e.g., need for home telecare; initiation of conventional measures in the case of high social risk) CG: usual care	6 months	Baseline 12 months
Mazya et al. [33], 2019 Sweden Parallel RCT	(Modified) Physical phenotype	Analysis of non-frail (robust/pre-frail) subsample of mixed (robust/pre-frail/frail) study population	N randomized Not reported separately for non-frail subsample N analyzed (IG) 80 (CG) 50 Total: 130	Not reported separately for non-frail subsample	Not reported separately for non-frail subsample	IG: CGA-based tailored care	IG: tailored CGA-based care delivered by an interdisciplinary team (nurse, social worker, pharmacist, physician, possibly physiotherapist, occupational therapist, and dietician) CG: usual care	24 months	Baseline 24 months
Monteserin et al. [34], 2010 Spain Parallel RCT	CGA	Analysis of non-frail subsample of mixed (non-frail/possibly frail) study population	N randomized (IG) 157 (CG) 178 Total: 335 N analyzed (IG) 113 (CG) 139 Total: 252	Not reported separately for non-frail subsample	Not reported separately for non-frail subsample	IG: CGA-based health promotion	IG: 45-min group session on health promotion, disease prevention, and self-care; booklet with health recommendations for older adults CG: usual care after CGA	45 min	Baseline 18 months
Serra-Prat et al. [35], 2017 Spain Parallel RCT	Physical phenotype	Pre-frail	N randomized^a^ (IG) 80 (CG) 92 Total: 172 N analyzed (IG) 61 (CG) 72 Total: 133	(IG) 77.9 (CG) 78.8	(IG) 48.7 (CG) 39.1	IG: exercise + nutrition	IG: 1. Home physical activity program with two main components: (i) aerobic exercise (walking outdoors 30–45 min per day ≥ 4 days per week) and (ii) 15 mixed exercises for arm strengthening, leg strengthening, balance, and coordination (20–25 min ≥ 4 days per week). 2. Screening with the Mini Nutritional Assessment Short-Form; participants at risk of malnutrition were referred to the Nutritional Unit for further management CG: usual care	12 months	Baseline 12 months
Upatising et al. [36], 2013 United States Parallel RCT	(Modified) Physical phenotype	Analysis of non-frail (robust/ pre-frail) subsample of mixed (robust/pre-frail/frail) study population	N randomized Not reported separately for non-frail subsample N analyzed (IG) 61 (CG) 75 Total: 136	Not reported separately for non-frail subsample	Not reported separately for non-frail subsample	IG: telemonitoring	IG: home telemonitoring with the Intel^®^ health guide (blood glucose level, blood pressure, oxygen saturation, pulse, weight). Measurements were based on an individualized protocol CG: usual care	12 months	Baseline 6 months
van Lieshout et al. [37], 2018 The Netherlands Parallel RCT	Groningen frailty indicator	Analysis of non-frail subsample of mixed (non-frail/frail) study population	N randomized Not reported separately for non-frail subsample N analyzed (IG) 81 (CG) 86 Total: 167	Not reported separately for non-frail subsample	Not reported separately for non-frail subsample	IG: multi-domain (medication review, nutrition, exercise, social)	IG: Supporting PRoactive lifestyle intervention in frailty and disability (SPRY) program: (1) Medication review with the Prescribing Optimization Method by a pharmacist. Consultation with primary care physician in the case adaptations of the medication were necessary. (2) Physical fitness sessions (two one-hour sessions per week over 12 weeks) including training in several daily activities (e.g.; walking stairs, shopping). (3) Training of social skills (five ~ 2.5 h weekly group meetings); assertiveness diary (4) Group information session with a dietician to increase awareness about healthy diet (2.5 h up to three times); diary of nutrition behavior CG: usual care	23 weeks	Baseline 12 months

*CG* Control group; *CGA* comprehensive geriatric assessment; *IG* intervention group; *N* number; *RCT* randomized controlled trial; *TID* three times a day

^a^Description of population at baseline (age, sex) is based on this sample

### Types of interventions and results of individual studies

Seven studies used exercise-related interventions [18, 29–32, 35, 37] and five studies nutritional interventions [28, 30, 32, 35, 37], either as a single intervention or combined with other types of interventions. Five studies investigated interventions with multiple components, such as a combination of exercise and nutrition [30, 35], CGA-based care tailored to the individual participants [33], and multi-domain interventions [32, 37]. One study evaluated the effect of telemonitoring on frailty incidence [36] and one study assessed the effect of a health promotion session after CGA [34]. The duration of interventions ranged from a single session to 24 months. Follow-up assessments were conducted between 8 weeks and 24 months after baseline. Five studies examined interventions with a duration of ≤ 16 weeks and analyzed frailty onset upon completion of the intervention. The effect estimates and 95% CIs for each trial included in the NMA of the frailty outcome are shown in online supplementary Table 4, and the narrative summaries of the results of the two remaining trials in online supplementary Table 5.

### Risk of bias

Table 2 lists the results of the risk of bias assessment for the individual studies. The majority of the studies were rated to have a low risk of bias arising from their randomization process (*n* = 8; 72.7%) and their measurement of the frailty outcome (*n* = 6; 54.5%). Nine studies (81.8%) raised some concerns or had a high risk of bias arising from missing outcome data. Eight out of the 11 included studies (72.7%) and six out of the nine studies included in the NMA (66.7%) were found to have a high overall risk of bias.

**Table 2:** Risk of bias for the frailty outcome in individual studies (Cochrane Risk of Bias 2 tool)

Study	Randomization process	Deviations from intended interventions	Missing outcome data	Measurement of the outcome	Selection of the reported result	Overall risk of bias
Badrasawi et al. 2016 [28]	Low	Some concerns	Some concerns	Low	High	High
Barrachina-Igual et al. 2021 [29]	Low	Low	Low	Some concerns	Some concerns	Some concerns
Biesek et al. 2021 [30]	Low	Low	Low	Low	Low	Low
Chen et al. 2020 [31]	Low	Some concerns	High	Low	Some concerns	High
Coelho-Júnior & Uchida 2021 [18]	Low	High	High	Some concerns	Some concerns	High
Gené Huguet et al. 2018 [32]	Some concerns	Some concerns	High	Some concerns	Some concerns	High
Mazya et al. 2019 [33]	Some concerns	Low	High	Low	Low	High
Monteserin et al. 2010 [34]	Low	High	High	Low	Some concerns	High
Serra-Prat et al. 2017 [35]	Low	Some concerns	Some concerns	Some concerns	Some concerns	Some concerns
Upatising et al. 2013 [36]	Low	High	High	Some concerns	Some concerns	High
van Lieshout et al. 2018 [37]	Some concerns	Some concerns	High	Low	Some concerns	High

### Network meta-analysis

#### Onset of frailty

**Fig. 2:**
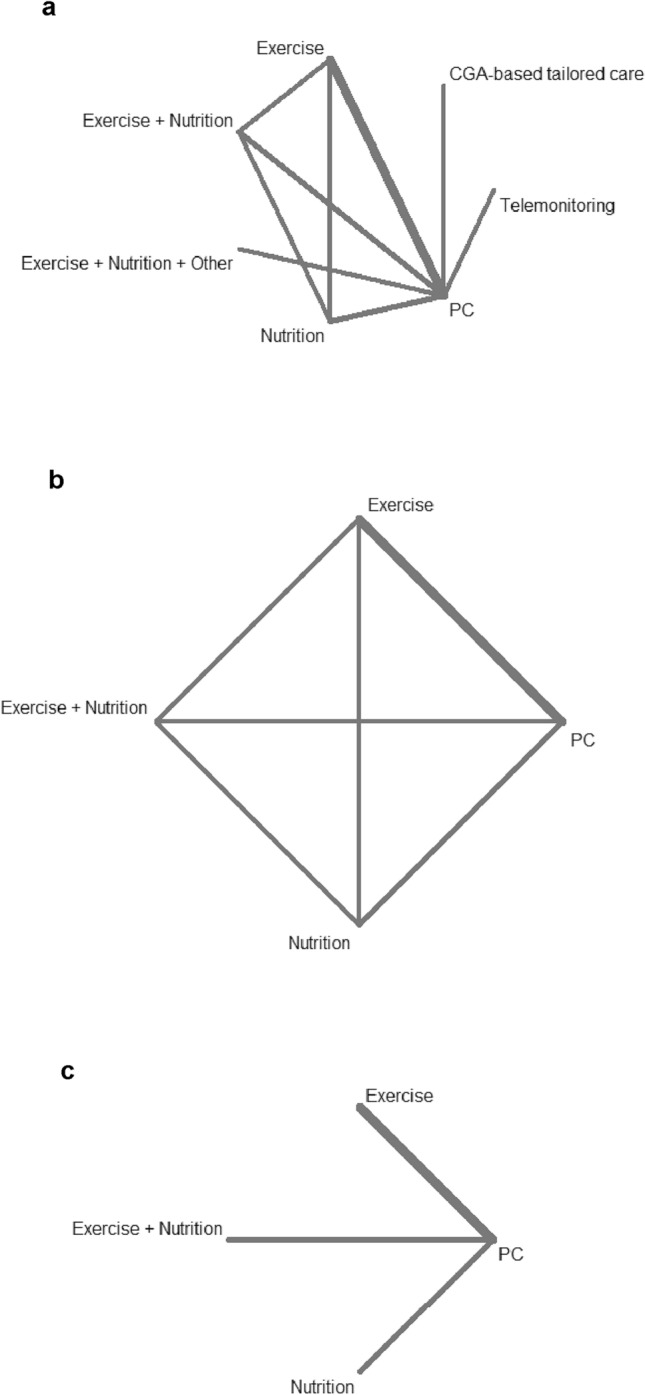
Geometry of the network for **a** the frailty outcome, **b** the frailty outcome in trials with a duration of intervention ≤ 16 weeks, and **c** for the gait speed outcome. *CGA* comprehensive geriatric assessment, *PC* control (placebo, usual care, minimally or likely ineffective control intervention)

**Fig. 3:**
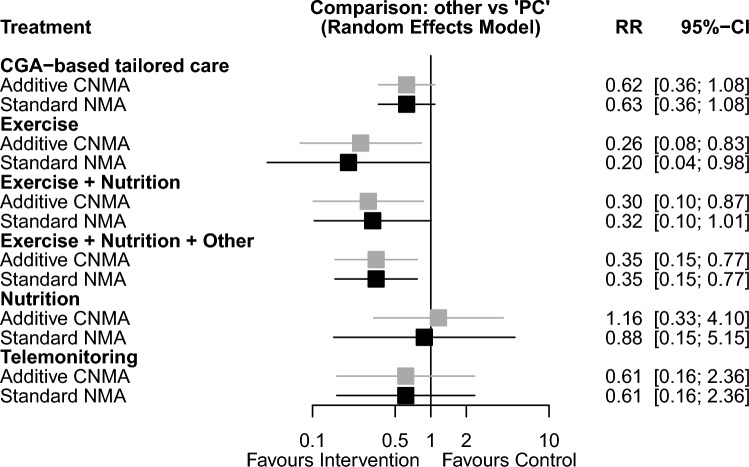
Relative risk of frailty onset for the different intervention types in the network meta-analysis (forest plot). *CGA* comprehensive geriatric assessment, *CI* confidence interval, *CNMA* component network meta-analysis, *NMA* network meta-analysis, *PC* control (placebo, usual care, minimally or likely ineffective control intervention), *RR* relative risk

All nine RCTs using the physical phenotype as a frailty outcome measure were included in an NMA (Fig. 2a), resulting in a total of 842 participants with 94 frailty events analyzed. In the CNMA of individual intervention components, exercise-based intervention (6 RCTs) was found to reduce the onset of frailty compared to placebo/control (RR 0.26, 95% CI 0.08; 0.83) (Fig. 3). There was no significant effect on frailty incidence for nutritional interventions (4 RCTs; RR 1.16, 95% CI 0.33; 4.10). The combination of exercise and nutrition again prevented frailty onset (RR 0.30, 95% CI 0.10; 0.87). The between-study variance was estimated to be *τ*^*2*^ = 0 and *I*^*2*^ = 0% [0.0%; 70.8%] and the Q statistics revealed neither heterogeneity nor inconsistency. The treatment effect estimates from the additive CNMA and standard NMA were similar and the additivity assumption seems justified (*p* = 0.66).

When interventions with a shorter duration (≤ 16 weeks) were analyzed separately (Fig. 2b), exercise (RR 0.23, 95% CI 0.06; 0.95) but not nutrition (RR 0.99, 95% CI 0.18; 5.42) showed a preventive effect on frailty onset (see online supplementary Fig. 1). The between-study variance was estimated to be *τ*^*2*^ = 0 and *I*^*2*^ = 0% [0.0%; 74.6%] and the Q statistics revealed neither heterogeneity nor inconsistency. The treatment effect estimates from the two models were similar and the additivity assumption seems justified (*p* = 0.73).

#### Gait speed

Gait speed was the most frequently used additional frailty instrument within the included trials, and four studies [18, 28, 31, 35] with a total of 281 participants using the physical phenotype as the main frailty assessment also reported gait speed as an outcome measure. Since the additivity assumptions could not be justified and the CNMA model revealed substantial heterogeneity (*τ*^*2*^ = 0.57 and *I*^*2*^ = 97.7% [95.6%; 98.8%], the results from the standard NMA are given (Fig. 2c). Exercise-based intervention improved gait speed (SMD 1.55, 95% CI 1.16, 1.95). Only one study reported results for nutrition and the combination of exercise and nutrition, respectively, hence no study results could be pooled in the NMA. The effect estimates and 95% CIs for the individual studies are shown in online supplementary Table 6 and the forest plot for this analysis in online supplementary Fig. 2. The between-study variance of the NMA model was estimated to be *τ*^*2*^ = 0.055 and *I*^*2*^ = 63.9% [0.0%; 91.7%].

### Additional outcome measures

Because the number of trials that reported on the secondary outcomes ADLs, IADLs, and health-related quality of life was low and the use of assessment instruments was heterogeneous, we did not perform a formal synthesis. Only one study gave a detailed description of adverse events without observing any statistically significant differences between groups [28].

### Investigation of publication bias

The comparison-adjusted funnel plot for the main outcome of incidental frailty revealed no small-study effects or other concerning effects in our study selection (see online supplementary Fig. 3). Where a comparison appeared only once in our analysis, it naturally fell in the center of the graph so no judgement on publication bias could be made. However, comparisons with multiple occurrences (e.g., Exercise: PC) showed a plausible spread around the overall effect.

### Characteristics of included study protocols

Online supplementary Table 7 lists the characteristics of the included study protocols. These protocols present the design of registered trials that have not yet published comprehensive results but might meet the eligibility criteria for this systematic review on frailty prevention. Out of the 22 study protocols identified, 11 reported using the Short Physical Performance Battery (SPPB) as an outcome measure for frailty, nine the physical phenotype of frailty, and three reported using the Clinical Frailty Scale. The trials are conducted in 13 different countries and across four continents. Nineteen out of the 22 trials use physical exercise or physical activity as an intervention component, while two evaluate coaching interventions to promote physical activity (see online supplementary Table 7).

## Discussion

This is the first systematic review with NMA to specifically examine the effectiveness of different types of interventions in the prevention of frailty onset. By selectively including non-frail populations and targeting frailty incidence as an outcome measure, this review adds to the existing evidence on the potential of different interventions to modify the course of the frailty pathway. Previous meta-analyses have reported intervention effects in mixed non-frail/frail populations [10, 38], evaluated the effect of a single type of intervention on frailty [39], or synthesized the results from cohort studies [40].

In our NMA, interventions that increased physical activity through physical exercise were found to significantly reduce the risk of frailty onset. This finding of a potentially beneficial effect of exercise and/or physical activity on frailty is in line with evidence from mixed non-frail/frail populations, indicating that physical exercise might be beneficial in the primary, secondary, and tertiary prevention of frailty. In their NMA, Negm and colleagues found that interventions based on physical activity significantly reduced frailty in mixed non-frail/frail populations [10]. Sun and colleagues identified physical activity, in particular resistance training, as the most effective non-pharmacological intervention to reduce frailty in populations with different frailty levels [13]. In a 2020 systematic review to inform the World Health Organization in the update of their physical activity guideline, Oliveira and colleagues conducted a meta-analysis with four studies that included mostly non-frail participants and a smaller number of frail participants [39]. This review concluded that interventions based on physical activity prevented frailty [39]. Moreover, in a 2022 meta-analysis of 10 cohort studies, a higher level of physical activity was associated with a reduced risk of frailty [40]. In a recent longitudinal analysis, participants who stopped exercising regularly experienced a more pronounced increase in their frailty index from midlife to older adulthood than those individuals who maintained a regular exercise regimen [41]. In addition, various types of exercise have been found to be beneficial in the management of constructs that are closely related to frailty, such as the treatment of sarcopenia [42] or the prevention of falls in older adults [43].

A recent NMA in populations with different levels of frailty found, that interventions that were based solely on nutrition were also effective in reducing frailty [13]. In contrast, nutritional interventions as a means to prevent frailty onset is not supported by our findings. In addition, we did not find an additive protective effect of exercise plus nutrition compared to exercise alone. Malnutrition has been identified as a risk factor for frailty [44], and evidence from cohort studies suggests that certain diets may be protective for frailty [45, 46]. A recent best-evidence summary stated that adherence to the Mediterranean dietary pattern may decrease the risk for frailty in older adults [47]. However, within our NMA, two of the four RCTs that examined the effectiveness of nutritional interventions had a duration and follow-up period of 12 weeks or less. It is conceivable that nutritional interventions require a longer period of time to be effective in preventing frailty, particularly in the absence of overt malnutrition. Moreover, the nutritional interventions were heterogeneous, with two of the four RCTs providing nutritional supplements, one trial providing nutritional counseling on the Mediterranean diet, and one trial providing malnutrition screening with a further referral of participants at risk of malnutrition for specialised management. In summary, the evidence base in this NMA appears insufficient to give a definite analysis of the role of nutritional interventions in frailty prevention.

However, outside of our systematic review, there is some additional evidence available from trials that included both middle-aged and older non-frail adults and examined the effect of specific nutritional supplements on physical performance or frailty incidence. A systematic review of RCTs observed a non-significant increase in gait speed after protein supplementation alone (mean duration 31 weeks) in non-frail community-dwelling adults aged > 50 years [48]. In an ancillary study of the Vitamin D and Omega-3 (VITAL) trial analyzing data from more than 25,000 participants, Orkaby and colleagues found that in adults aged ≥ 50 years (men) or aged ≥ 55 years (women) supplementation with 2000 IU/d vitamin D_3_ or 1 g/d omega-3 fatty acids did not reduce frailty incidence (frailty index, physical phenotype) during the 5-year follow-up time-frame [49]. Recent data suggest that the findings of this latter RCT are also applicable to the older robust population [50]. Overall, this research appears to support the negative results of our NMA with regard to the effectiveness of nutritional interventions in frailty prevention.

Given the manifold risk factors for and dimensions of frailty, one would expect that, besides physical exercise, multi-component interventions and CGA-based interventions might prove to be particularly effective. These types of interventions do not only cover the physical and nutritional aspects of frailty but honor the holistic concept of frailty, e.g., by also targeting the social facets of this geriatric syndrome. In our NMA, we could not demonstrate evidence for the effectiveness of such resource-intensive strategies due to the low number of included trials examining these types of interventions.

Although our findings appear to be supported by previous research on the modifying effects of exercise on frailty, the results of our NMA should be interpreted with caution. Owing to the limited number of studies, the statistical heterogeneity of the model could not be estimated with confidence. Tau2 was estimated to be 0, which may indicate an absence of heterogeneity, but more likely indicates that it was not possible to calculate this parameter properly. The overall risk of bias was rated as high for six out of the nine studies included in the NMA. This was mostly due to a high risk of bias in the domain of missing outcome data. Sample sizes of the eligible studies were generally small and the number of frailty events was low, and we judged loss to follow-up due to unspecified reasons or exclusion based on insufficient adherence as potentially related to the frailty status of the participants. Moreover, apart from the results on the risk of bias tool, other limitations of the individual studies should be considered. The majority of the studies included in the NMA used slightly modified versions of the physical phenotype of frailty. Modified versions of the frailty phenotype are common in research studies but might influence how many individuals are identified as frail by this frailty measure [51]. The majority of the studies assessed the frailty outcome directly after the end of the intervention. Therefore, future research will have to address whether intervention delivery will need to be continuous or intermittent to maintain preventive effects over a longer period of time.

The strengths of this systematic review include the comprehensive literature search with a detailed full-text screening of more than 1000 articles and the exact, i.e., quantifiable and reproducible, identification of the frailty status of the participants during the selection process. We applied predefined cut-points for frailty to specifically collect the evidence for the non-frail older population. Interventions to prevent and treat frailty are a rapidly growing field [13], and we were able to identify 22 protocols without published full-text data and to provide a detailed overview of this sizeable number of trials that are ongoing or in the process of analyzing results.

There are also limitations of this review that have to be mentioned. We explicitly targeted frailty incidence as an outcome measure which could have favored frailty assessments that are commonly presented with absolute cut-points (e.g., the physical phenotype) over those assessments that tend to be presented as continuous data (e.g., frailty index, SPPB, gait speed, timed up and go test). To reduce heterogeneity, we only performed our NMA with the nine out of the 11 included trials that assessed the frailty outcome using the physical phenotype. Low physical activity is one of the five frailty criteria on the physical phenotype, and it is unclear, how this could have biased the results towards the interventions based on physical exercise. Only one [28] of the trials within the NMA used a frailty index as an additional frailty assessment. Accordingly, an analysis examining potential differences in results between the two main frailty models (physical phenotype, frailty index) was not possible. Owing to the low number of studies included in this systematic review, we did not perform additional analysis on the preventive effect of different types of physical exercise (e.g., resistance training, aerobic training, balance).

Based on the results of our NMA, exercise-based interventions appear to be effective in frailty prevention. Given the severe consequences of frailty, the implementation of classes providing exercise to community-dwelling older adults offers the potential to be beneficial on both an individual and a societal level. In view of the limited resources within the healthcare sector and the large number of non-frail older adults, these interventions should initially focus on those individuals at immediate risk of frailty, i.e., pre-frail older adults. In addition, involving non-specialists may help to make exercise interventions available to and affordable for as many older persons as possible.

This review and NMA was not able to define which type, frequency, and intensity of physical exercise would be most appropriate for the prevention of frailty onset. Moreover, frailty goes beyond physical fitness and activity. The nutritional and psychosocial and potentially iatrogenic aspects (e.g., medication optimization) of frailty might be particularly challenging to modify and require more complex and long-run interventions. Future research needs to also design studies that embrace these challenges.

## Conclusion

The results of this systematic review and NMA suggest, that interventions based on physical exercise could be effective in preventing the onset of frailty in robust and pre-frail older adults. The data analyzed in this systematic review came from a limited number of studies with a small number of participants and often a high risk of bias. Larger intervention studies with frailty incidence as a distinct outcome measure are needed to solidify the evidence on the prevention of frailty onset.

## Supplementary Information

Below is the link to the electronic supplementary material.

Supplementary file1 (DOCX 69 KB)

## Data Availability

Not applicable.
