# Supplementary material for: Interventions to prevent the onset of frailty in adults aged 60 and older (PRAE-Frail): a systematic review and network meta-analysis
Source: Eur Geriatr Med. 2024 Jul 26;15(5):1169–85. doi: 10.1007/s41999-024-01013-x (PMC11614966; doi:10.1007/s41999-024-01013-x)
Supplement: Supplementary file 1 — Supplementary file1 (DOCX 69 KB) [file 41999_2024_1013_MOESM1_ESM.docx]

**Online Supplementary Material**

**Journal:**

European Geriatric Medicine

**Title:**

Interventions to Prevent the Onset of Frailty in Adults Aged 60 and Older (PRAE-Frail):

A Systematic Review and Network Meta-Analysis

**Authors and author affiliations:**

Annette Eidam^1^, Jane Durga^1^, Jürgen M. Bauer^1,2^, Samuel Zimmermann^3^, Johannes A. Vey^3^, Kilian Rapp^4^, Michael Schwenk^5^, Matteo Cesari^6^, Petra Benzinger^1,7^

^1^ Center for Geriatric Medicine, Heidelberg University Hospital, Agaplesion Bethanien Hospital Heidelberg, Heidelberg, Germany

^2^ Network Aging Research (NAR), Heidelberg University, Heidelberg, Germany

^3^ Institute of Medical Biometry, Heidelberg University, Heidelberg, Germany

^4^ Department of Clinical Gerontology, Robert-Bosch-Hospital, Stuttgart, Germany

^5^ Department of Sport Science, Human Performance Research Centre, University of Konstanz, Konstanz, Germany

^6^ Department of Clinical Sciences and Community Health, University of Milan, Milan, Italy

^7^ Institute of Health and Generations, Faculty of Social and Health Studies, University of Applied Sciences Kempten, Kempten, Germany

**Corresponding author:**

Annette Eidam

Center for Geriatric Medicine, Heidelberg University Hospital,

Agaplesion Bethanien Hospital Heidelberg

Rohrbacher Straße 149, 69126 Heidelberg

Tel: +49-6221-319-1795

E-mail: [Annette.Eidam@agaplesion.de](mailto:Annette.Eidam@agaplesion.de)

**Supplementary Table S1:** PRISMA NMA Checklist of Items to Include When Reporting A Systematic Review Involving a Network Meta-analysis [18].

| **Section/Topic** | **Item #** | **Checklist Item** | **Reported on Page #** |
| --- | --- | --- | --- |
| **TITLE** |  |  |  |
| Title | 1 | Identify the report as a systematic review *incorporating a network meta-analysis (or related form of meta-analysis).* | ***1*** |
|  |  |  |  |
| **ABSTRACT** |  |  |  |
| Structured summary | 2 | Provide a structured summary including, as applicable:  **Background:** main objectives  **Methods:** data sources; study eligibility criteria, participants, and interventions; study appraisal; and *synthesis methods, such as network meta-analysis.*  **Results:** number of studies and participants identified; summary estimates with corresponding confidence/credible intervals; *treatment rankings may also be discussed. Authors may choose to summarize pairwise comparisons against a chosen treatment included in their analyses for brevity.*  **Discussion/Conclusions:** limitations; conclusions and implications of findings.  **Other:** primary source of funding; systematic review registration number with registry name. | 3 |
|  |  |  |  |
| **INTRODUCTION** |  |  |  |
| Rationale | 3 | Describe the rationale for the review in the context of what is already known*, including mention of why a network meta-analysis has been conducted.* | ***4-5*** |
| Objectives | 4 | Provide an explicit statement of questions being addressed, with reference to participants, interventions, comparisons, outcomes, and study design (PICOS). | 5 |
|  |  |  |  |
| **METHODS** |  |  |  |
| Protocol and registration | 5 | Indicate whether a review protocol exists and if and where it can be accessed (e.g., Web address); and, if available, provide registration information, including registration number. | 5 |
| Eligibility criteria | 6 | Specify study characteristics (e.g., PICOS, length of follow-up) and report characteristics (e.g., years considered, language, publication status) used as criteria for eligibility, giving rationale. *Clearly describe eligible treatments included in the treatment network, and note whether any have been clustered or merged into the same node (with justification).* | ***5-7*** |
| Information sources | 7 | Describe all information sources (e.g., databases with dates of coverage, contact with study authors to identify additional studies) in the search and date last searched. | 7-8 |
| Search | 8 | Present full electronic search strategy for at least one database, including any limits used, such that it could be repeated. | 8, Table S3 |
| Study selection | 9 | State the process for selecting studies (i.e., screening, eligibility, included in systematic review, and, if applicable, included in the meta-analysis). | 8 |
| Data collection process | 10 | Describe method of data extraction from reports (e.g., piloted forms, independently, in duplicate) and any processes for obtaining and confirming data from investigators. | 8 |
| Data items | 11 | List and define all variables for which data were sought (e.g., PICOS, funding sources) and any assumptions and simplifications made. | 8-9 |
| **Geometry of the network** | **S1** | Describe methods used to explore the geometry of the treatment network under study and potential biases related to it. This should include how the evidence base has been graphically summarized for presentation, and what characteristics were compiled and used to describe the evidence base to readers. | ***9-10*** |
| Risk of bias within individual studies | 12 | Describe methods used for assessing risk of bias of individual studies (including specification of whether this was done at the study or outcome level), and how this information is to be used in any data synthesis. | 9 |
| Summary measures | 13 | State the principal summary measures (e.g., risk ratio, difference in means). *Also describe the use of additional summary measures assessed, such as treatment rankings and surface under the cumulative ranking curve (SUCRA) values, as well as modified approaches used to present summary findings from meta-analyses.* | 9-10 |
| Planned methods of analysis | 14 | Describe the methods of handling data and combining results of studies for each network meta-analysis. This should include, but not be limited to:   - *Handling of multi-arm trials;* - *Selection of variance structure;* - *Selection of prior distributions in Bayesian analyses; and* - *Assessment of model fit.* | 9-10 |
| **Assessment of Inconsistency** | **S2** | Describe the statistical methods used to evaluate the agreement of direct and indirect evidence in the treatment network(s) studied. Describe efforts taken to address its presence when found. | 10 |
| Risk of bias across studies | 15 | Specify any assessment of risk of bias that may affect the cumulative evidence (e.g., publication bias, selective reporting within studies). | **10** |
| Additional analyses | 16 | Describe methods of additional analyses if done, indicating which were pre-specified. This may include, but not be limited to, the following:   - Sensitivity or subgroup analyses; - Meta-regression analyses; - *Alternative formulations of the treatment network; and* - *Use of alternative prior distributions for Bayesian analyses (if applicable).* | ***10*** |
|  |  |  |  |
| **RESULTS†** |  |  |  |
| Study selection | 17 | Give numbers of studies screened, assessed for eligibility, and included in the review, with reasons for exclusions at each stage, ideally with a flow diagram. | 11, Figure 1 |
| **Presentation of network structure** | **S3** | Provide a network graph of the included studies to enable visualization of the geometry of the treatment network. | ***Figure 2*** |
| **Summary of network geometry** | **S4** | Provide a brief overview of characteristics of the treatment network. This may include commentary on the abundance of trials and randomized patients for the different interventions and pairwise comparisons in the network, gaps of evidence in the treatment network, and potential biases reflected by the network structure. | ***12-13*** |
| Study characteristics | 18 | For each study, present characteristics for which data were extracted (e.g., study size, PICOS, follow-up period) and provide the citations. | 11-12, Table 1 |
| Risk of bias within studies | 19 | Present data on risk of bias of each study and, if available, any outcome level assessment. | 12, Table 2 |
| Results of individual studies | 20 | For all outcomes considered (benefits or harms), present, for each study: 1) simple summary data for each intervention group, and 2) effect estimates and confidence intervals. *Modified approaches may be needed to deal with information from larger networks.* | ***Table S4, Table S5*** |
| Synthesis of results | 21 | Present results of each meta-analysis done, including confidence/credible intervals. *In larger networks, authors may focus on comparisons versus a particular comparator (e.g. placebo or standard care), with full findings presented in an appendix. League tables and forest plots may be considered to summarize pairwise comparisons.* If additional summary measures were explored (such as treatment rankings), these should also be presented. | ***12-13, Figure 3, Figure S1, Figure S2*** |
| **Exploration for inconsistency** | **S5** | Describe results from investigations of inconsistency. This may include such information as measures of model fit to compare consistency and inconsistency models, *P* values from statistical tests, or summary of inconsistency estimates from different parts of the treatment network. | ***12-13*** |
| Risk of bias across studies | 22 | Present results of any assessment of risk of bias across studies for the evidence base being studied. | 14, Figure S3 |
| Results of additional analyses | 23 | Give results of additional analyses, if done (e.g., sensitivity or subgroup analyses, meta-regression analyses*, alternative network geometries studied, alternative choice of prior distributions for Bayesian analyses,* and so forth). | ***13*** |
|  |  |  |  |
| **DISCUSSION** |  |  |  |
| Summary of evidence | 24 | Summarize the main findings, including the strength of evidence for each main outcome; consider their relevance to key groups (e.g., healthcare providers, users, and policy-makers). | 14-18 |
| Limitations | 25 | Discuss limitations at study and outcome level (e.g., risk of bias), and at review level (e.g., incomplete retrieval of identified research, reporting bias). *Comment on the validity of the assumptions, such as transitivity and consistency. Comment on any concerns regarding network geometry (e.g., avoidance of certain comparisons).* | 16-17; 17-18 |
| Conclusions | 26 | Provide a general interpretation of the results in the context of other evidence, and implications for future research. | 18-19 |
|  |  |  |  |
| **FUNDING** |  |  |  |
| Funding | 27 | Describe sources of funding for the systematic review and other support (e.g., supply of data); role of funders for the systematic review. This should also include information regarding whether funding has been received from manufacturers of treatments in the network and/or whether some of the authors are content experts with professional conflicts of interest that could affect use of treatments in the network. | ***19*** |

PICOS = population, intervention, comparators, outcomes, study design.

* Text in italics indicateS wording specific to reporting of network meta-analyses that has been added to guidance from the PRISMA statement.

† Authors may wish to plan for use of appendices to present all relevant information in full detail for items in this section.

**Supplementary Table S2:** List of frailty assessment instruments considered valid in this systematic review, including their hierarchy ranks and cut-points for frailty.

| **Instrument** | **Hierarchy** | **Cut-points** | **Notes** |
| --- | --- | --- | --- |
| Clinical frailty scale [1, 2] | 1 | Frailty ≥ 5 [3] |  |
| Comprehensive geriatric assessment [4] | 1 | Based on clinical judgement |  |
| Comprehensive geriatric assessment frailty index [5] | 1 | See Frailty index |  |
| Easy-Care [6] | 1 | Frailty ≥ 60 [7] |  |
| Edmonton Frail Scale [8] | 2 | Frailty ≥ 7 [3] |  |
| Fatigue, resistance, ambulation, illness, loss of weight (FRAIL) scale [9] | 4 | Frailty ≥ 3  Pre-frail = 1-2 [3] |  |
| Frailty index // Frailty index of accumulated deficits [10] | 1 | Frailty > 0.25 [3] |  |
| Fried and colleagues’ physical phenotype of frailty [11] | 3 | Frailty ≥ 3  Pre-frail = 1-2 [3] |  |
| Frailty risk score / Prognostic risk score [12] | 1 | Frailty ≥ 56 points [12] |  |
| Frailty trait scale [13] | 3 | Frailty ≥ 50 [13] |  |
| Gait speed [14] | 6 | Frailty < 0.8 m/s [7] | Highest preference of physical measures [15]  Measured over 2-10 m at usual pace |
| Gérontopôle frailty screening tool [16] | 1 | Based on clinical judgment [3] |  |
| Groningen frailty indicator [17] | 3 | Frailty ≥ 4 [3] |  |
| Identification of seniors at risk [18] | 4 | Frailty (patients at risk of adverse outcomes) ≥ 2 [19] |  |
| Kihon checklist [20] | 1 | Frailty > 0.25 [3] |  |
| Multidimensional prognostic index [21] | 1 | Frailty > 0.66  Pre-frail = 0.34-0.66 [3] |  |
| PRISMA-7 [22] | 4 | Frailty ≥ 3 [3] |  |
| Rapid geriatric assessment [23] | 2 | Based on clinical judgement [23] |  |
| Self-rated health deficits index [24] | 4 | See Frailty index |  |
| Sherbrooke postal questionnaire [25] | 4 | Frailty ≥ 2 [3] |  |
| Short physical performance battery [26] | 5 | Frailty ≤ 7 [27] |  |
| Study of osteoporotic fracture index / SOF frailty criteria [28] | 4 | Frailty ≥ 2  Pre-frail = 1 [3] |  |
| Tilburg frailty indicator [29] | 4 | Frailty ≥ 5 [3] |  |
| Timed up and go / get up and go [30] | 7 | Frailty ≥ 10 s [31] | Measured over 3 m / 10 ft at usual pace |
| Vulnerable elders survey [32] | 4 | Frailty ≥ 3 [33] |  |

**Notes:** The list of frailty assessments was derived from a recent review [3] and a clinical practice guideline for the management of frailty [34]. Differing from these references, we did not consider grip strength (inconsistent cut-points for frailty in the literature) and the G8 (special disease population) as valid frailty assessments for the purposes of this systematic review. The hierarchy ranges from 1 = highest ranked frailty instrument to 7 = lowest ranked frailty instrument.

**References**

1. Rockwood K, Song X, MacKnight C, Bergman H, Hogan DB, McDowell I et al. A global clinical measure of fitness and frailty in elderly people. CMAJ. 2005;173(5):489-95. doi:10.1503/cmaj.050051.

2. Rockwood K, Theou O. Using the Clinical Frailty Scale in Allocating Scarce Health Care Resources. Can Geriatr J. 2020;23(3):210-5. doi:10.5770/cgj.23.463.

3. Dent E, Kowal P, Hoogendijk EO. Frailty measurement in research and clinical practice: A review. Eur J Intern Med. 2016;31:3-10. doi:10.1016/j.ejim.2016.03.007.

4. Cesari M, Gambassi G, van Kan GA, Vellas B. The frailty phenotype and the frailty index: different instruments for different purposes. Age Ageing. 2014;43(1):10-2. doi:10.1093/ageing/aft160.

5. Rockwood K, Rockwood MR, Mitnitski A. Physiological redundancy in older adults in relation to the change with age in the slope of a frailty index. J Am Geriatr Soc. 2010;58(2):318-23. doi:10.1111/j.1532-5415.2009.02667.x.

6. Craig C, Chadborn N, Sands G, Tuomainen H, Gladman J. Systematic review of EASY-care needs assessment for community-dwelling older people. Age Ageing. 2015;44(4):559-65. doi:10.1093/ageing/afv050.

7. Brefka S, Dallmeier D, Muhlbauer V, von Arnim CAF, Bollig C, Onder G et al. A Proposal for the Retrospective Identification and Categorization of Older People With Functional Impairments in Scientific Studies-Recommendations of the Medication and Quality of Life in Frail Older Persons (MedQoL) Research Group. J Am Med Dir Assoc. 2019;20(2):138-46. doi:10.1016/j.jamda.2018.11.008.

8. Rolfson DB, Majumdar SR, Tsuyuki RT, Tahir A, Rockwood K. Validity and reliability of the Edmonton Frail Scale. Age Ageing. 2006;35(5):526-9. doi:10.1093/ageing/afl041.

9. Morley JE, Malmstrom TK, Miller DK. A simple frailty questionnaire (FRAIL) predicts outcomes in middle aged African Americans. J Nutr Health Aging. 2012;16(7):601-8. doi:10.1007/s12603-012-0084-2.

10. Mitnitski AB, Mogilner AJ, Rockwood K. Accumulation of deficits as a proxy measure of aging. ScientificWorldJournal. 2001;1:323-36. doi:10.1100/tsw.2001.58.

11. Fried LP, Tangen CM, Walston J, Newman AB, Hirsch C, Gottdiener J et al. Frailty in older adults: evidence for a phenotype. J Gerontol A Biol Sci Med Sci. 2001;56(3):M146-56. doi:10.1093/gerona/56.3.m146.

12. Pijpers E, Ferreira I, van de Laar RJ, Stehouwer CD, Nieuwenhuijzen Kruseman AC. Predicting mortality of psychogeriatric patients: a simple prognostic frailty risk score. Postgrad Med J. 2009;85(1007):464-9. doi:10.1136/pgmj.2008.073353.

13. Garcia-Garcia FJ, Carcaillon L, Fernandez-Tresguerres J, Alfaro A, Larrion JL, Castillo C et al. A new operational definition of frailty: the Frailty Trait Scale. J Am Med Dir Assoc. 2014;15(5):371 e7- e13. doi:10.1016/j.jamda.2014.01.004.

14. Stanaway FF, Gnjidic D, Blyth FM, Le Couteur DG, Naganathan V, Waite L et al. How fast does the Grim Reaper walk? Receiver operating characteristics curve analysis in healthy men aged 70 and over. BMJ. 2011;343:d7679. doi:10.1136/bmj.d7679.

15. Hoogendijk EO, van Kan GA, Guyonnet S, Vellas B, Cesari M. Components of the Frailty Phenotype in Relation to the Frailty Index: Results From the Toulouse Frailty Platform. J Am Med Dir Assoc. 2015;16(10):855-9. doi:10.1016/j.jamda.2015.04.007.

16. Vellas B, Balardy L, Gillette-Guyonnet S, Abellan Van Kan G, Ghisolfi-Marque A, Subra J et al. Looking for frailty in community-dwelling older persons: the Gerontopole Frailty Screening Tool (GFST). J Nutr Health Aging. 2013;17(7):629-31. doi:10.1007/s12603-013-0363-6.

17. Peters LL, Boter H, Buskens E, Slaets JP. Measurement properties of the Groningen Frailty Indicator in home-dwelling and institutionalized elderly people. J Am Med Dir Assoc. 2012;13(6):546-51. doi:10.1016/j.jamda.2012.04.007.

18. McCusker J, Bellavance F, Cardin S, Trepanier S, Verdon J, Ardman O. Detection of older people at increased risk of adverse health outcomes after an emergency visit: the ISAR screening tool. J Am Geriatr Soc. 1999;47(10):1229-37. doi:10.1111/j.1532-5415.1999.tb05204.x.

19. Yao JL, Fang J, Lou QQ, Anderson RM. A systematic review of the identification of seniors at risk (ISAR) tool for the prediction of adverse outcome in elderly patients seen in the emergency department. Int J Clin Exp Med. 2015;8(4):4778-86.

20. Satake S, Senda K, Hong YJ, Miura H, Endo H, Sakurai T et al. Validity of the Kihon Checklist for assessing frailty status. Geriatr Gerontol Int. 2016;16(6):709-15. doi:10.1111/ggi.12543.

21. Pilotto A, Ferrucci L, Franceschi M, D'Ambrosio LP, Scarcelli C, Cascavilla L et al. Development and validation of a multidimensional prognostic index for one-year mortality from comprehensive geriatric assessment in hospitalized older patients. Rejuvenation Res. 2008;11(1):151-61. doi:10.1089/rej.2007.0569.

22. Raiche M, Hebert R, Dubois MF. PRISMA-7: a case-finding tool to identify older adults with moderate to severe disabilities. Arch Gerontol Geriatr. 2008;47(1):9-18. doi:10.1016/j.archger.2007.06.004.

23. Morley JE, Adams EV. Rapid Geriatric Assessment. J Am Med Dir Assoc. 2015;16(10):808-12. doi:10.1016/j.jamda.2015.08.004.

24. Lucicesare A, Hubbard RE, Searle SD, Rockwood K. An index of self-rated health deficits in relation to frailty and adverse outcomes in older adults. Aging Clin Exp Res. 2010;22(3):255-60. doi:10.1007/BF03324805.

25. Hebert R, Bravo G, Korner-Bitensky N, Voyer L. Predictive validity of a postal questionnaire for screening community-dwelling elderly individuals at risk of functional decline. Age Ageing. 1996;25(2):159-67. doi:10.1093/ageing/25.2.159.

26. Guralnik JM, Simonsick EM, Ferrucci L, Glynn RJ, Berkman LF, Blazer DG et al. A short physical performance battery assessing lower extremity function: association with self-reported disability and prediction of mortality and nursing home admission. J Gerontol. 1994;49(2):M85-94. doi:10.1093/geronj/49.2.m85.

27. European Medicines Agency. Reflection paper on physical frailty: instruments for baseline characterisation of older populations in clinical trials. 2018. Accessed 13 Dec 2022.

28. Ensrud KE, Ewing SK, Taylor BC, Fink HA, Stone KL, Cauley JA et al. Frailty and risk of falls, fracture, and mortality in older women: the study of osteoporotic fractures. J Gerontol A Biol Sci Med Sci. 2007;62(7):744-51. doi:10.1093/gerona/62.7.744.

29. Gobbens RJ, van Assen MA, Luijkx KG, Wijnen-Sponselee MT, Schols JM. The Tilburg Frailty Indicator: psychometric properties. J Am Med Dir Assoc. 2010;11(5):344-55. doi:10.1016/j.jamda.2009.11.003.

30. Podsiadlo D, Richardson S. The timed "Up & Go": a test of basic functional mobility for frail elderly persons. J Am Geriatr Soc. 1991;39(2):142-8. doi:10.1111/j.1532-5415.1991.tb01616.x.

31. British Geriatrics Society. Recognising frailty. https://www.bgs.org.uk/resources/recognising-frailty. Accessed 13 Dec 2022.

32. Chapman MD, Le BH, Gorelik A. The Vulnerable Elders Survey and its prognostic relationship to survival in an older community-based palliative population. BMJ Support Palliat Care. 2013;3(3):335-42. doi:10.1136/bmjspcare-2012-000386.

33. Buta BJ, Walston JD, Godino JG, Park M, Kalyani RR, Xue QL et al. Frailty assessment instruments: Systematic characterization of the uses and contexts of highly-cited instruments. Ageing Res Rev. 2016;26:53-61. doi:10.1016/j.arr.2015.12.003.

34. Dent E, Lien C, Lim WS, Wong WC, Wong CH, Ng TP et al. The Asia-Pacific Clinical Practice Guidelines for the Management of Frailty. J Am Med Dir Assoc. 2017;18(7):564-75. doi:10.1016/j.jamda.2017.04.018.

**Supplementary Table S3:** Search strategy for the PubMed search.

| # | **Search Term** |
| --- | --- |
| 1 | ((“randomized controlled trial“[Publication Type]) OR (“controlled clinical trial”[Publication Type]) OR (randomized[Title/Abstract]) OR (randomised[Title/Abstract]) OR (randomly[Title/Abstract]) OR (trial[Title]) OR (“clinical trials as topic”[MeSH Major Topic: noexp])) |
| 2 | ((frail*[Text Word]) OR (comprehensive geriatric assessment[Text Word]) OR (Easy Care[Text Word]) OR (EASYcare[Text Word]) OR (Fried* phenotype[Text Word]) OR (prognostic risk score[Text Word]) OR (gait speed[Text Word]) OR (walking speed[Text Word]) OR (grip strength[Text Word]) OR (identification of seniors at risk[Text Word]) OR (kihon checklist[Text Word]) OR (multidimensional prognostic index[Text Word]) OR (PRISMA-7[Text Word]) OR (rapid geriatric assessment[Text Word]) OR (self-rated health deficit* index[Text Word]) OR (sherbrooke postal questionnaire[Text Word]) OR (short physical performance battery[Text Word]) OR (study of osteoporotic fracture index[Text Word]) OR (“timed up and go”[Text Word]) OR (“get up and go”[Text Word]) OR (vulnerable elder survey[Text Word]) OR (vulnerable elders survey[Text Word])) |
| 3 | #1 AND #2 |

Filter: Humans

**Supplementary Table S4:** Study and arm specific effect estimates for the frailty outcome (RR with 95 % CI adjusted for multiarm studies).

| **RCT (Author, year)** | **Comparison**  **(treatment 1 vs. treatment 2)** | **N analyzed treatment 1** | **N frailty events**  **treatment 1** | **N analyzed treatment 2** | **N frailty events**  **treatment 2** | **RR [95 %-CI] for frailty** |
| --- | --- | --- | --- | --- | --- | --- |
| Badrasawi et al [28], 2016 | Nutrition vs. PC | 26 | 0 | 24 | 0 | 0.92 [0.02-44.83] |
| Barrachina-Igual et al [29], 2021 | Exercise vs. PC | 23 | 0 | 20 | 4 | 0.10 [0.01-1.69] |
| Biesek et al [30], 2021 | Exercise vs. Exercise + Nutrition | 30 | 0 | 16 | 0 | 0.54 [0.00-1540.53] |
|  | Exercise vs. Nutrition | 30 | 0 | 18 | 1 | 0.20 [0.00-17.67] |
|  | Exercise vs. PC | 30 | 0 | 15 | 1 | 0.17 [0.00-14.25] |
|  | Exercise + Nutrition vs. Nutrition | 16 | 0 | 18 | 1 | 0.37 [0.00-31.65] |
|  | Exercise + Nutrition vs. PC | 16 | 0 | 15 | 1 | 0.31 [0.00-25.52] |
|  | Nutrition vs. PC | 18 | 1 | 15 | 1 | 0.84 [0.07-9.94] |
| Chen et al [31], 2020 | Exercise vs. PC | 33 | 0 | 33 | 1 | 0.33 [0.01-7.89] |
| Coelho-Júnior & Uchida [18], 2021 | Exercise vs. PC | 22 | 0 | 10 | 0 | 0.47 [0.01-21.95] |
| Gené Huguet et al [32], 2018 | Exercise + Nutrition + Other vs. PC | 85 | 7 | 88 | 21 | 0.35 [0.15-0.77] |
| Mazya et al [33], 2019 | CGA-based tailored care vs. PC | 80 | 18 | 50 | 18 | 0.62 [0.36-1.08] |
| Serra-Prat et al [35], 2017 | Exercise + Nutrition vs. PC | 61 | 3 | 72 | 11 | 0.32 [0.09-1.1] |
| Upatising et al [36], 2013 | Telemonitoring vs. PC | 61 | 3 | 75 | 6 | 0.61 [0.16-2.36] |

*CGA: comprehensive geriatric assessment; CI: confidence interval; N: number; PC: control group (placebo, usual care, minimally or likely ineffective control intervention) within the respective trial; RCT: randomized controlled trial; RR: relative risk.*

**Supplementary Table S5:** Results for the frailty outcome – trials not included in the network meta-analysis.

| **Author, year** | **Main frailty assessment** | **Population** | **Sample size** | **Type of intervention** | **Duration of intervention** | **Follow-up assessment for frailty** | **Results for main frailty assessment** |
| --- | --- | --- | --- | --- | --- | --- | --- |
| Monteserin et al [34], 2010 | CGA | Analysis of non-frail subsample of mixed (non-frail/  possibly frail) study population | *N randomized*  IG) 157 CG) 178 Total: 335  *N analyzed*  IG) 113 CG) 139 Total: 252 | IG: CGA-based health promotion | 45 min | 18 months | Progression from "not at risk of frailty" to "at risk of frailty" at follow-up was higher in the CG (33.8 %) compared with the IG (20.4 %) (p = 0.023). |
| van Lieshout et al [37], 2018 | Groningen frailty indicator | Analysis of non-frail subsample of mixed (non-frail/frail) study population | *N randomized*  not reported separately for non-frail subsample  *N analyzed*  IG) 81 CG) 86 Total: 167 | IG: Multi-domain (medication review, nutrition, exercise, social) | 23 weeks | 12 months | No statistical difference at 12 months between IG and CG: OR for frailty (adjusted for frailty at baseline) 0.99, 95 % CI 0.54 to 1.82, p = 0.97). |

*CG: control group; CGA: comprehensive geriatric assessment; CI: confidence interval; IG: intervention group; N: number; OR: odds ratio.*


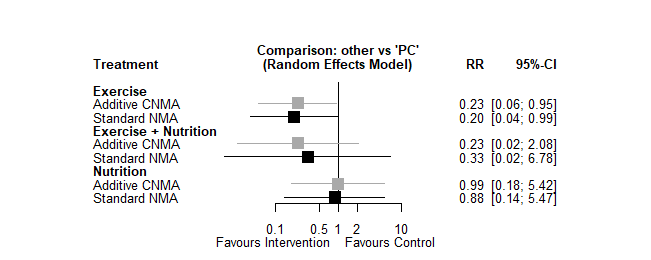


**Supplementary Figure S1:** Relative risk of frailty onset for the different intervention types in the network meta-analysis in trials with a duration of intervention ≤ 16 weeks (forest plot). *CI* confidence interval, *CNMA* component network meta-analysis, *NMA* network meta-analysis, *PC* control (placebo, usual care, minimally or likely ineffective control intervention), *RR* relative risk

**Supplementary Table S6:** Study and arm specific effect estimates for the gait speed outcome.

| **RCT (Author, year)** | **Treatment 1** | **Treatment 2** | **SMD [95 %-CI]** |
| --- | --- | --- | --- |
| Badrasawi et al [28], 2016 | Nutrition | PC | 0.25 [0.03-0.47] |
| Chen et al [31], 2020 | Exercise | PC | 1.71 [1.51-1.91] |
| Coelho-Júnior & Uchida [18], 2021 | Exercise | PC | 1.30 [0.85-1.74] |
| Serra-Prat et al [35], 2017 | Exercise + Nutrition | PC | 0.50 [0.42-0.57] |

*CI: confidence interval; PC: control group (placebo, usual care, minimally or likely ineffective control intervention) within the respective trial; RCT: randomized controlled trial; SMD: standardized mean difference.*


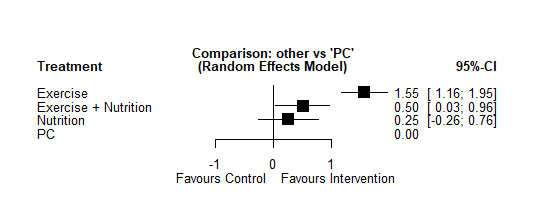


**Supplementary Figure S2:** Standardized mean differences of the comparisons between the different intervention types versus the comparator node for the gait speed outcome (forest plot). *CI* confidence interval, *PC* control (placebo, usual care, minimally or likely ineffective control intervention)

**
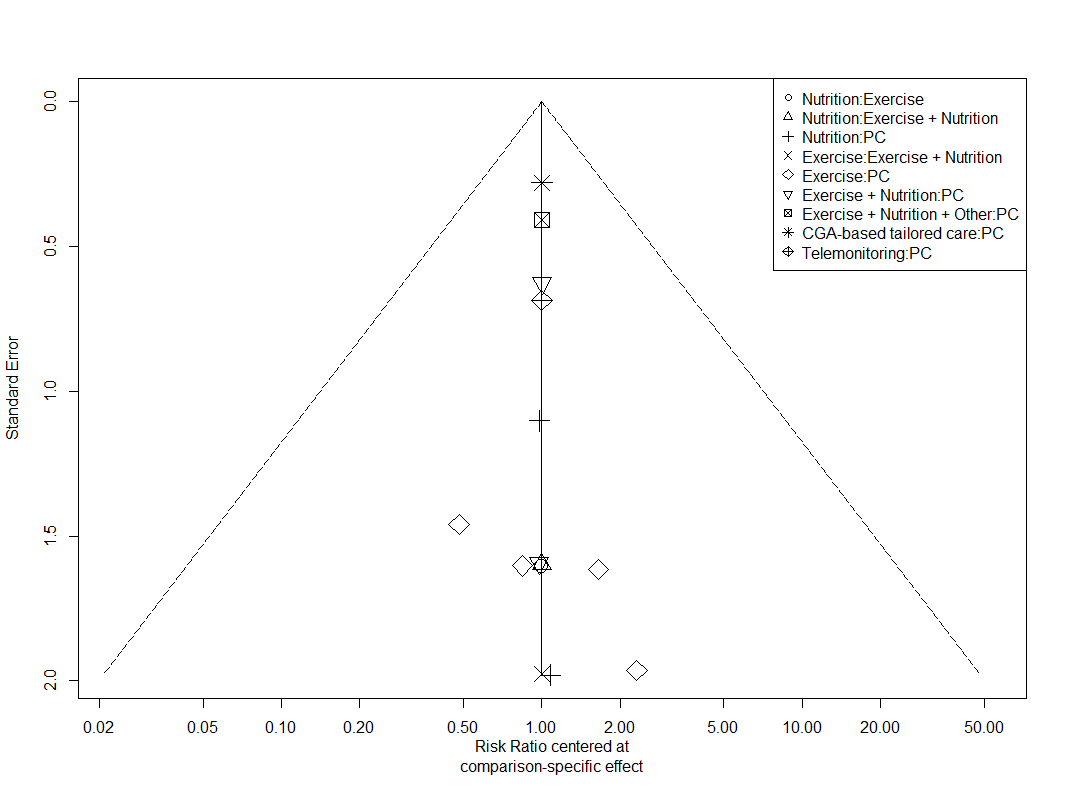
**

**Supplementary Figure S3:** Investigation of publication bias in the network meta-analysis – comparison adjusted funnel plot for the main outcome of incidental frailty. *CGA* comprehensive geriatric assessment, *PC* control (placebo, usual care, minimally or likely ineffective control intervention)

**Supplementary Table S7:** Characteristics of included study protocols. These protocols present the design of registered trials that have not yet published comprehensive results, but might meet the eligibility criteria for this systematic review on frailty prevention.

| **Author, year** | **Sample size** | **Duration** | **Study population** | **Intervention** | **Frailty outcome** | **Country** | **Trial registration number** |
| --- | --- | --- | --- | --- | --- | --- | --- |
| Borges et al [1],  2021 | 230 | varied | ≥ 60 years Recently discharged from hospital and on waiting list for physical therapy | Telerehabilitation via smartphone app (individualized exercises for training resistance, balance and a daily activity of the participant’s choice) | TUG | Brazil | RBR-9243v7 |
| Castell et al [2],  2019 | 190 | 12-week intervention with 12 months follow-up | > 70 years Pre-frail (Fried) | Multicomponent physical activity program | Fried SPPB | Spain | NCT03568084 |
| Daly et al [3],  2015 | 280 | 18 months | ≥ 65 years At increased risk for falls | Dual-task functional power training | Gait speed TUG | Australia | ACTRN12613001161718 |
| Ekdahl et al [4],  2018 | 450 | 24 months | ≥ 75 years ≥ 3 visits to emergency care unit in prior 18 months | CGA-based care | SPPB Fried CFS | Sweden | NCT02923843 |
| Fairhall et al [5], 2015 | 230 | 12 months | ≥ 70 years  Pre-frail (Fried) | Individually tailored interdisciplinary and multifactorial intervention targeting identified Fried frailty characteristics and additional problems identified during geriatric assessment (several possible components including exercise, nutritional and psychological management) | Fried  SPPB  Gait speed | Australia | ACTRN12613000043730 |
| Giné-Garriga et al [6],  2017 | 1338 | 22 months  (4-month intervention + 18 months follow-up) | ≥ 65 years | 1. Exercise referral scheme with self-management strategies 2. Exercise referral scheme | SPPB | Spain Denmark United Kingdom Germany | NCT02629666 |
| Grede et al [7],  2021 | 345 | 12 months  (6-month intervention + 6 months follow-up) | ≥ 65 years SPPB score < 9 Recruited from the community or nursing homes | Walking program with volunteer | SPPB CFS | Germany | DRKS00015188 |
| Haeger et al [8],  2022 | 254 | 12 months | ≥ 75 years | Tablet-assisted motivational counseling related to mobility | Fried | Germany | DRKS00025230 |
| Jofré-Saldía et al [9], 2021 | 102 | 27 weeks | 60-80 years | Progressive multicomponent exercise program | SPPB TUG Gait speed | Spain | NCT04118478 |
| Khow et al [10],  2018 | 80 | 24 weeks  (12-week intervention + 12 weeks follow-up) | ≥ 65 years FRAIL scale score ≥ 1 | Personalized coaching intervention for physical activity | Gait speed | Australia | ACTRN12617001186347 |
| Majumdar et al [11], 2021 | 250 | 24 weeks | ≥ 60-80 years | Yoga-based program (physical activity, relaxation, dietary advice, group lectures on yogic philosophy/social relationships) | Gait speed | India | CTRI/2021/02/031373 |
| McCaskill et al [12], 2019 | 40 | 8 weeks | ≥ 65 years | DVD-based home exercise program | SPPB | United States | NCT03580551 |
| Mohd Suffian et al [13], 2020 | 60 | 6 months  (3-month intervention + 3 months follow-up) | ≥ 60 years Pre-frail (Fried) | Multicomponent exercise program, nutrition education | Fried SPPB | Malaysia | NCT04327544 |
| Murukesu et al [14], 2020 | 72 | 6 months | ≥ 60 years With cognitive pre-frailty or cognitive frailty (Fried pre-frail/frail and Clinical Dementia Rating Scale score 0.5) | Multi-domain: multicomponent exercise, dietary counseling, cognitive training, psychosocial support | Fried Gait speed TUG | Malaysia | ACTRN12619001055190 |
| Rivas-Ruiz et al [15], 2020 | 200 | 12 months | ≥ 70 years SPPB score < 10 | Multi-domain: exercise (muscle-strengthening exercises, walks), nutritional care, medication review | SPPB | Spain | ISRCTN17143761 |
| Shen et al [16],  2016 | 92 | 20 weeks  (8-week intervention + 12 weeks follow-up) | ≥ 65 years Sarcopenic obese | Three intervention groups:  Nutrition Exercise Nutrition + Exercise | SPPB Gait speed | China | ChiCTR-IOR-15007501 |
| Sugimoto et al [17], 2021 | 500 | 18 months | 65-85 years With mild cognitive impairment | Multi-domain:  exercise, nutrition counseling, cognitive training | CGA (including Fried + gait speed) | Japan | UMIN000038671 |
| Teljigovic et al [18], 2020 | 177 | 12-week intervention with 12-month follow-up | ≥ 65 years Referred to rehabilitation center | Two intervention groups: Nutrition (protein supplementation) Exercise + Nutrition (protein supplementation) | PRISMA-7 TFI TUG | Denmark | NCT04091308 |
| Uzor et al [19],  2013 | 48 | 12 weeks | ≥ 65 years Experienced a fall in the 12 months prior | Two intervention groups:  Movement visualization during home rehabilitation Multimodal movement games during home rehabilitation | Gait speed TUG | United Kingdom | ISRCTN79967470 |
| van Gameren et al [20], 2021 | 256 | 14-week intervention with 12-month follow-up | ≥ 65 years Pre-frail and robust (Fried) | Fall prevention program with Tai Chi-based exercises and fall prevention education | Fried Gait speed TUG | The Netherlands | NL9248 |
| Wei et al [21],  2021 | 114 | 24 weeks (12-week intervention + 12 weeks follow-up) | ≥ 65 years With dynapenia | Exercise (flexi-bar training) | TUG Gait speed | China | ISRCTN14316668 |
| Werner et al [22],  2022 | 400 | 12 months | ≥ 70 years Mixed frailty status (CFS score 4-6) | Home-based exercises, facultative counseling services (e.g., nutrition) | CFS Fried SPPB | Germany | DRKS00024638 |

*CFS: Clinical Frailty Scale; CGA: comprehensive geriatric assessment; Fried: Fried and colleagues’ physical phenotype of frailty; SPPB: Short Physical Performance Battery; TFI: Tilburg frailty indicator; TUG: Timed Up and Go.*

**References**

1. Borges PRT, Resende RA, Dias JF, Mancini MC, Sampaio RF. Telerehabilitation program for older adults on a waiting list for physical therapy after hospital discharge: study protocol for a pragmatic randomized trial protocol. Trials. 2021;22(1):445. doi:10.1186/s13063-021-05387-2.

2. Castell MV, Gutierrez-Misis A, Sanchez-Martinez M, Prieto MA, Moreno B, Nunez S et al. Effectiveness of an intervention in multicomponent exercise in primary care to improve frailty parameters in patients over 70 years of age (MEFAP-project), a randomised clinical trial: rationale and study design. BMC Geriatr. 2019;19(1):25. doi:10.1186/s12877-018-1024-8.

3. Daly RM, Duckham RL, Tait JL, Rantalainen T, Nowson CA, Taaffe DR et al. Effectiveness of dual-task functional power training for preventing falls in older people: study protocol for a cluster randomised controlled trial. Trials. 2015;16:120. doi:10.1186/s13063-015-0652-y.

4. Ekdahl AW, Axmon A, Sandberg M, Steen Carlsson K. Is care based on comprehensive geriatric assessment with mobile teams better than usual care? A study protocol of a randomised controlled trial (The GerMoT study). BMJ Open. 2018;8(10):e023969. doi:10.1136/bmjopen-2018-023969.

5. Fairhall N, Kurrle SE, Sherrington C, Lord SR, Lockwood K, John B et al. Effectiveness of a multifactorial intervention on preventing development of frailty in pre-frail older people: study protocol for a randomised controlled trial. BMJ Open. 2015;5(2):e007091. doi:10.1136/bmjopen-2014-007091.

6. Gine-Garriga M, Coll-Planas L, Guerra M, Domingo A, Roque M, Caserotti P et al. The SITLESS project: exercise referral schemes enhanced by self-management strategies to battle sedentary behaviour in older adults: study protocol for a randomised controlled trial. Trials. 2017;18(1):221. doi:10.1186/s13063-017-1956-x.

7. Grede N, Rieckert A, Muth J, Steinbuck J, Weissbach S, Schneider A et al. A volunteer-supported walking programme to improve physical function in older people (the POWER Study): study protocol for a randomised controlled trial. BMC Geriatr. 2021;21(1):45. doi:10.1186/s12877-020-01988-9.

8. Haeger C, Mumken SA, O'Sullivan JL, Spang RP, Voigt-Antons JN, Stockburger M et al. Mobility enhancement among older adults 75 + in rural areas: Study protocol of the MOBILE randomized controlled trial. BMC Geriatr. 2022;22(1):65. doi:10.1186/s12877-021-02739-0.

9. Jofre-Saldia E, Villalobos-Gorigoitia A, Gea-Garcia G. Effects of multicomponent exercise program with progressive phases on functional capacity, fitness, quality of life, dual-task and physiological variables in older adults: Randomized controlled trial protocol. Rev Esp Geriatr Gerontol. 2021;56(5):272-8. doi:10.1016/j.regg.2021.04.006.

10. Khow KS, Dollard J, Bray K, Smyth C, Chehade M, Theou O et al. A randomized controlled feasibility study to evaluate the effects of a goal-setting coaching intervention using feedback from an accelerometer on sedentary time in older people at risk of falls (SMART-MOVE): a study protocol. Pilot Feasibility Stud. 2018;4:173. doi:10.1186/s40814-018-0366-5.

11. Majumdar V, Snigdha A, Manjunath NK, Nagarathna R, Mavathur R, Singh A et al. Study protocol for yoga-based lifestyle intervention for healthy ageing phenotype in the older adults (yHAP): a two-armed, waitlist randomised controlled trial with multiple primary outcomes. BMJ Open. 2021;11(9):e051209. doi:10.1136/bmjopen-2021-051209.

12. McCaskill GM, Clay OJ, Motl RW, Ball KK. Older Veterans EmpoweRed To Use Regular Exercise (OVERTURE) II: Design and methods of a randomized controlled trial among older veterans with chronic health conditions. Contemp Clin Trials Commun. 2019;15:100395. doi:10.1016/j.conctc.2019.100395.

13. Mohd Suffian NI, Adznam SN, Abu Saad H, Chan YM, Ibrahim Z, Omar N et al. Frailty Intervention through Nutrition Education and Exercise (FINE). A Health Promotion Intervention to Prevent Frailty and Improve Frailty Status among Pre-Frail Elderly-A Study Protocol of a Cluster Randomized Controlled Trial. Nutrients. 2020;12(9). doi:10.3390/nu12092758.

14. Murukesu RR, Singh DKA, Shahar S, Subramaniam P. A Multi-Domain Intervention Protocol for the Potential Reversal of Cognitive Frailty: "WE-RISE" Randomized Controlled Trial. Front Public Health. 2020;8:471. doi:10.3389/fpubh.2020.00471.

15. Rivas-Ruiz F, Machon M, Mateo-Abad M, Contreras-Fernandez E, Guell C, Baro-Rodriguez L et al. Tackling frailty at primary care: evaluation of the effectiveness of a multicomponent intervention through a randomised controlled trial: study protocol. BMJ Open. 2020;10(2):e034591. doi:10.1136/bmjopen-2019-034591.

16. Shen SS, Chu JJ, Cheng L, Zeng XK, He T, Xu LY et al. Effects of a nutrition plus exercise programme on physical function in sarcopenic obese elderly people: study protocol for a randomised controlled trial. BMJ Open. 2016;6(9):e012140. doi:10.1136/bmjopen-2016-012140.

17. Sugimoto T, Sakurai T, Akatsu H, Doi T, Fujiwara Y, Hirakawa A et al. The Japan-Multimodal Intervention Trial for Prevention of Dementia (J-MINT): The Study Protocol for an 18-Month, Multicenter, Randomized, Controlled Trial. J Prev Alzheimers Dis. 2021;8(4):465-76. doi:10.14283/jpad.2021.29.

18. Teljigovic S, Sogaard K, Sandal LF, Dalager T, Nielsen NO, Sjogaard G et al. Individualised physical exercise training and enhanced protein intake in older citizens during municipality-based rehabilitation: protocol for a randomised controlled trial. BMJ Open. 2020;10(11):e041605. doi:10.1136/bmjopen-2020-041605.

19. Uzor S, Baillie L, Skelton DA, Rowe PJ. Falls prevention advice and visual feedback to those at risk of falling: study protocol for a pilot randomized controlled trial. Trials. 2013;14:79. doi:10.1186/1745-6215-14-79.

20. van Gameren M, Bossen D, Bosmans JE, Visser B, Frazer SWT, Pijnappels M. The (cost-)effectiveness of an implemented fall prevention intervention on falls and fall-related injuries among community-dwelling older adults with an increased risk of falls: protocol for the in balance randomized controlled trial. BMC Geriatr. 2021;21(1):381. doi:10.1186/s12877-021-02334-3.

21. Wei N, Wang X, Chen L, Lyu M. Effects of flexi-bar training on muscle strength and physical performance in older people with dynapenia: the protocol of a randomised controlled trial. BMJ Open. 2021;11(8):e048629. doi:10.1136/bmjopen-2021-048629.

22. Werner C, Wolf-Belala N, Nerz C, Abel B, Braun T, Gruneberg C et al. A multifactorial interdisciplinary intervention to prevent functional and mobility decline for more participation in (pre-)frail community-dwelling older adults (PromeTheus): study protocol for a multicenter randomized controlled trial. BMC Geriatr. 2022;22(1):124. doi:10.1186/s12877-022-02783-4.
